# Gender Trends in First Authorship of Academic Publications Related to Wolff-Parkinson-White Syndrome

**DOI:** 10.7759/cureus.47208

**Published:** 2023-10-17

**Authors:** Nadia Djahanshahi, Sheethal Seelamanthula, FNU Shubhangi, Nikhil Sai Jagarlamudi, Arushi Dhawan, Vellanki Vidya Spandana

**Affiliations:** 1 Medicine, St. George's University, True Blue, GRD; 2 Medical Education, Sri Padmavathi Medical College for Women, Sri Venkateswara Institute of Medical Sciences, Tirupati, IND; 3 Internal Medicine, Nalanda Medical College and Hospital, Patna, IND; 4 Internal Medicine, NRI Medical College, Guntur, IND; 5 Internal Medicine, California Institute of Behavioral Neurosciences and Psychology, Fairfield, USA; 6 Internal Medicine, Navodaya Medical College, Raichur, IND

**Keywords:** country-specific contribution, publication, financial status, research, gender equality, tachycardia, wpw syndrome

## Abstract

Wolff-Parkinson-White (WPW) syndrome is a congenital cardiac preexcitation syndrome that arises from abnormal cardiac electrical conduction through an accessory pathway and results in symptomatic and life-threatening arrhythmias. The aim of this study is to analyze the patterns of gender representation among first-author publications concerning “Wolf-Parkinson-White syndrome” within the PubMed-indexed publications from “January 1, 1973, to December 31, 2022,” based on country and year. On May 9, 2023, bibliometric analysis was performed. The phrase "(Wolf-Parkinson-White Syndrome)" was looked up in PubMed. It covered articles released between January 1, 1973, and December 31, 2022. Articles accepted in the year 2022 and published in Pubmed in 2023 were included in the study. A total of 138 articles were considered and included in our analysis. Among these articles, 29 (21.01%) were authored by females, while 109 (78.99%) were authored by males. To conclude, this research study reveals a rising trend of females in lead authorship roles within the field of cardiac arrhythmia research. However, it remains evident that there is a significant gender gap, with male researchers still outnumbering their female counterparts.

## Introduction and background

Education, healthcare, employment, and social standards are just a few of the areas where gender has a big impact on society. Understanding gender patterns can provide insight into the inequities, inequalities, and changing dynamics of society. Researchers can help to identify areas where interventions or policy changes may be required to promote gender equality and socioeconomic advancement by looking at gender patterns. Academic medical advancement is mostly dependent on yield and the calculated external impact of one's scholarly effort. The publication of original research in prestigious journals and requests from editors to offer scientific assessments of an individual's published research are two examples of objective measurements of the impact of one's work [1]. Researchers can advance the academic conversation, expand on current information, and get fresh perspectives on gender dynamics by analyzing gender trends. Research on gender trends can help institutions, organizations, and policymakers make decisions that are supported by facts. It offers a framework for creating initiatives, programs, and laws that tackle gender-based issues, encourage inclusivity, and guarantee equitable opportunity for people of all genders.

Wolff-Parkinson-White (WPW) syndrome is a congenital cardiac preexcitation syndrome that arises from abnormal cardiac electrical conduction through an accessory pathway and results in symptomatic and life-threatening arrhythmias. Classic ECG findings of WPW pattern or preexcitation are represented by a sinus rhythm, short PR interval, and prolonged QRS with an initial slurring upstroke also known as the delta wave. However, WPW syndrome denotes an ECG pattern consistent with the described findings above and along with the coexistence of a tachyarrhythmia and clinical symptoms of tachycardia such as palpitations, episodic lightheadedness, presyncope, syncope, or even cardiac arrest [2]. WPW syndrome is associated with significant morbidity and mortality. Although it is rare, some patients' initial presentation may be ventricular fibrillation or sudden cardiac death [3]. WPW syndrome is being explored as a research topic to investigate gender trends among first authors due to the paramount concern of potential sudden death associated with the condition. It is important to understand that the risk of sudden death is a driving factor in the study and management of WPW syndrome [4].

By focusing on the first authors, the researchers aimed to understand the gender distribution and potential disparities at the forefront of research and publication in WPW syndrome. Although there are now more female doctors and trainees in cardiology than ever before, it is still unclear if the share of women in academic cardiology literature is increasing at the same rate [5]. This exploration can provide valuable insights for policymakers, funding agencies, and academic institutions to implement targeted interventions that promote gender equity, improve opportunities for underrepresented groups, and foster a more inclusive research environment.

This research paper aims to offer an analysis of PubMed-indexed publications on "Wolf-Parkinson-White syndrome" and analyze the gender distribution of first authors. The objectives include evaluating the gender distribution of first authors in the identified publications, investigating the geographical representation of first authors, analyzing country-specific trends, and using statistical methods to forecast how the gender distribution of first authors may potentially change over time.

By achieving these objectives, this research aims to provide insights into gender representation and country-specific contributions to WPW syndrome research. The findings can contribute to promoting diversity and inclusivity in the scientific community.

## Review

On May 9, 2023, we performed a bibliometric analysis. The phrase "(Wolf-Parkinson-White syndrome)" was looked up in PubMed. It covered articles released between January 1, 1973, and December 31, 2022. Since just a few publications of four months accessible, publications from the year 2023 were removed. Articles accepted in the year 2022 and published and appeared on Pubmed in 2023 were included in the study.

The total number of entries was equally divided among all six authors. In order to conduct the analysis, the full names, author rankings, and countries of the authors found in PubMed during the time frame in question were extracted. The full names of the authors were determined from the selected manuscripts. The order in which the authors were listed in the manuscript was used to establish author rank. The authors' countries were confirmed based on the institutional affiliation of the respective authors. Namsor (Namsor, Versailles, France), a program used to automatically match genders, full names, and countries of authors, was utilized to find out the genders [6]. Statistical analysis was done using the R software (The R Foundation, Vienna, Austria), the autoregressive integrated moving average (ARIMA) model, and graphs prepared using DataWrapper (Datawrapper, Berlin, Germany).

**Figure 1:**
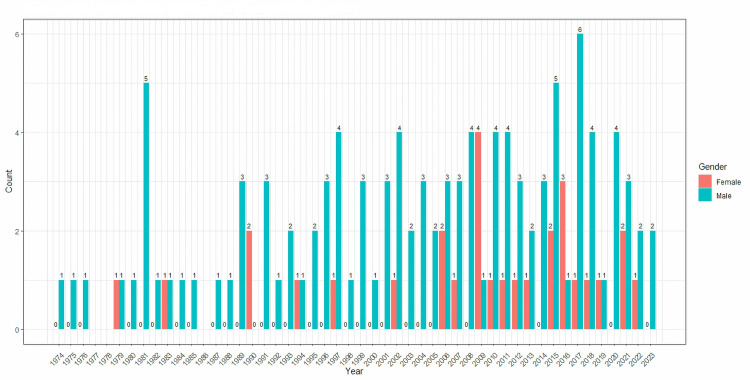
Total number of male and female first authors each year

A total of 138 articles were considered and included in our analysis. Among these articles, 29 (21.01%) were authored by females, while 109 (78.99%) were authored by males. Figure 1 presents the distribution of male and female authors over the years. The peak number of female first-author publications on WPW syndrome occurred in 2008, with a total of four publications. On the other hand, the highest number of male first-author publications on WPW syndrome was observed in 2017, with a total of six publications.

**Figure 2:**
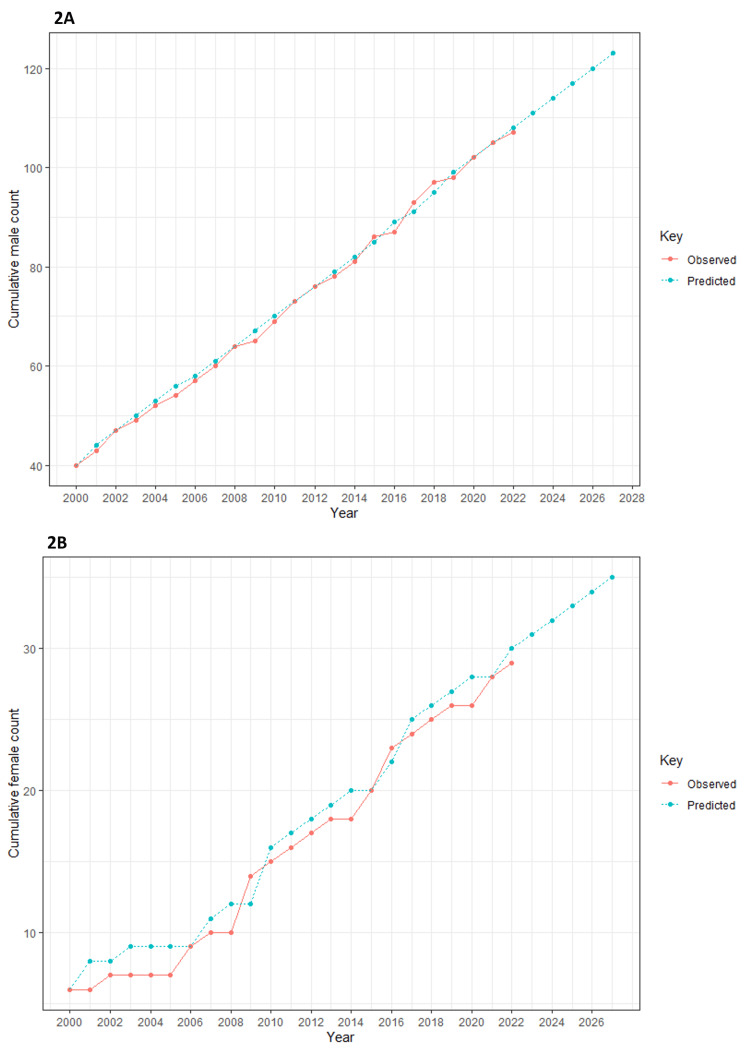
The projected number of publications by male and female first authors for the next 10 years (from 2000 to 2027) Projection is made using the ARIMA model

Figure 2 displays the projected number of publications by male and female first authors for the next 10 years (from 2000 to 2027). According to the ARIMA model, in 2027, we anticipate approximately 125 publications by male first authors and around 35 publications by female first authors. It is important to note that the year 2023 was excluded from the model due to incomplete data.

**Figure 3:**
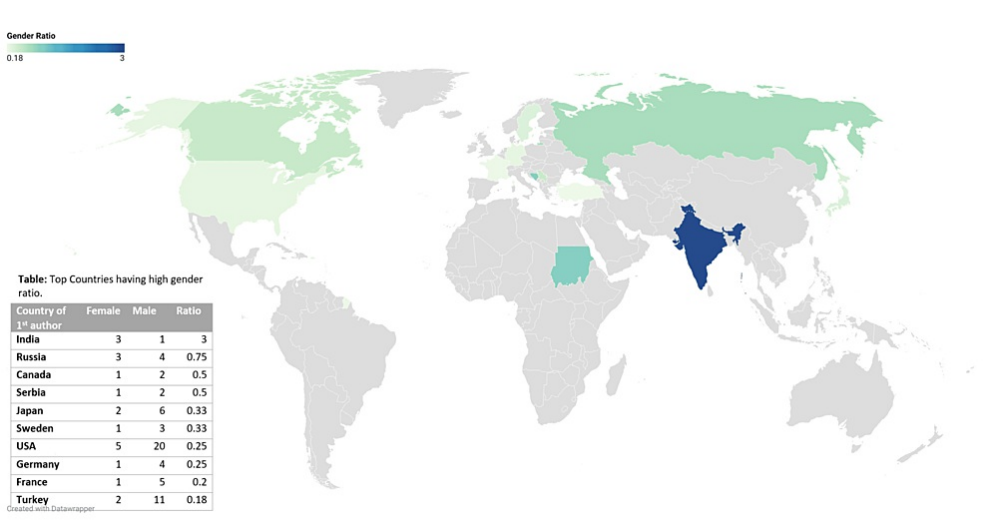
Gender trends in publications (1974-2023) based on country

Figure 3 illustrates the female-to-male ratio across various countries. The gender ratios ranged from 0.18 to 3, with a value of 1 indicating equal publishing rates between genders and a value of 3 signifying a 3:1 ratio favoring females. The highest gender ratio was observed in India, followed by Sudan. In contrast, France, Turkey, and the United States had the lowest gender ratios.

In Table 1, we present the top journals with favorable gender ratios. Kardiologiia exhibited the most favorable outcome, with a female-to-male first-author ratio of 1:3 (0.33). The journal Cardiol Young also displayed a positive gender ratio, with a female-to-male author ratio of 1:2 (0.5). We conducted the Fisher's exact test to determine if there was any association between first author gender and country variables. The statistical analysis revealed a p-value of 0.147 (p<0.05 indicates statistical significance), indicating no significant association between gender and country.

**Table 1:** Top journals having a favorable gender ratio

Journal/book	Female	Male	Ratio
Anadolu Kardiyol Derg	1	1	1
Arch Pediatr	1	1	1
Herzschrittmacherther Elektrophysiol	1	1	1
Int J Cardiol	1	1	1
Pediatr Cardiol	2	2	1
Srp Arh Celok Lek	1	1	1
Cardiol Young	1	2	0.5
Kardiologiia	1	3	0.33

Our study focused on the first authors of the articles published on the topic of WPW syndrome over the last 50 years and found that out of the total of 138 identified articles spanning from January 1, 1973, to December 31, 2022, the representation of female first authors was notably low, accounting for only 21.01% (29) of the 138 authors, while male authors constituted 78.99% (109) of the total.

Though the study showed an increase in the number of female authors in recent years, the first few decades had an alarmingly low amount of representation, with only seven female authors noted in three decades, and a steady increase in female participation was observed from 2006. A similar result was seen in a study done by Foley et al. on gender equality in academic gastroenterology. From a total of 865 abstracts over 40 years, they recorded only 13.8% female first authors and 10.5% female senior authors, and the number of female authors has tripled since the 2000s [7]. A review by Nigel et al., on the authorship demographics in the American Journal of Cardiology showed evidence that even when women represent more than half of the medical students, the number of female cardiologists constitute as low as 13% of all United States in 2013 [8]. They recorded an increase in the total number of female first authorships from 3% to only 23% in 58 years. This explains the initial plateau and the slow increase in the cumulative graph of the number of female authors in our study compared to the linear increase in male authors.

When analyzed on the basis of the geographical origin of the first authors of WPW syndrome, similar to the study by Morgan et al., authors from high-income countries (HIC) published significantly more than the authors from middle-income countries (MIC) and lower-income countries (LIC) [9]. On examining the gender distribution, unlike the study by Morgan et al., where there was an increase in the percentage of female authorship from LIC 25.4%, MIC 29.7%, and HIC 37.5%, the number of women authors was very low compared to male authors irrespective of HIC or LIC, with LIC 25%, MIC 28.5%, and HIC 17.3%. On the contrary, the study conducted by Ravi et al. recorded a greater number of publications from MIC and LIC when compared to HIC [10]. However, even when 70% of the global healthcare workforce in low- or middle-income countries (LMICs) was made up of women, the male representation still exceeded the female authorship. Surprisingly, when the gender ratio of female-to-male authors was calculated in our study, India, a MIC, had the highest ratio. This can be compared to the United States, an HIC, and Turkey, an upper MIC which has a lower gender ratio. These findings require further investigations focusing specifically on gender equity in LMICs.

The articles published in top journals when specifically observed show that, although the acceptance rate is low, there is no gender disparity in accepting the publications in the majority of them. This shows that the attempt of academic journals to attain greater gender equality by enacting multiple strategies and improving female representation is inclining toward the positive side of the success spectrum [11].

A forecasting analysis was done to estimate the increase in the number of authors for the next five years, up to 2027, using the ARIMA model for both male and female authors (Figure 2A-2b) [12]. In statistical analysis, we found no significant change in the gender gap even after five years. In a similar study by Rickard et al., the number of female authorships is comparatively high in medical affiliations rather than in surgical affiliations, and gender equity was observed in a few medical-affiliated publications [13]. They did a forecasting analysis for three decades and observed that females may comprise about 83% of the first authors in pediatric urology literature, a medical affiliation, which is reassuring but does not comply with our study. However, when they considered the present scenario, even with an overall increase in the number of authorships in both genders, globally, the number of female authors still lags behind, similar to our study.

Multiple studies focused their investigation on the reasons for the diminished female representation in academic research. A comparative study by Ross et al. stated that women are less likely to be named in a given article or given any patent rights. Scientific contributions by women are less likely to be recognized [14]. The obligatory role of women in domestic and marital responsibilities and reproductive labor is found as one of the main barriers to female accomplishments in both clinical and academic aspects [11].

The gender gap is present in most of the scientific fields and almost all career stages. Richter et al. found that women physicians are less likely to be promoted to higher ranks like associate professor, full professor, or department chair positions. Thus, an underrepresentation of women has been observed among residency program directors, who are role models and sponsors for career advancement, and on editorial boards of medical journals, who determine which authors will have their work published, thus, leading to impaired possible mentorship to female authors [15]. A survey conducted by Rimmer et al. in the United Kingdom found that two-thirds of female consultant cardiologists (62%) have experienced discrimination at work, which is three times the proportion (20%) of men. The basis of discrimination ranges from gender and parenting to ethnicity. Many physicians reported sexual harassment and comments [16].

Low financial status is another important barrier to be considered. This accounts for not only women authorship but also all the authors from MIC and LIC who cannot afford research and publication. More journals should adopt and implement the concept of “fee-waivers” for women and LMICs, thus encouraging authors to publish from all over the world and not just from privileged countries.

A study by Nakayama et al. in Japan claimed that patients cared for by female cardiologists and general interns have fewer 30-day mortality and readmission rates than those cared for by their male counterparts. This is because female physicians practice evidence-based, guideline-directed medicine and communicate with other experts and probably communicate well with the patients and, thus, are successful in providing better patient-centered preventive care than male cardiologists [17]. This emphasizes the need to implement measures that aid female physicians to focus more on both clinical practice and academic domains. These include workplace nurseries for child-rearing physicians and workload management by appointing more young physicians and interns.

Gender inequity has been a prominent issue in a variety of fields even after women have proven to be efficient and productive over the generations. Our study, although focusing on a specific topic in cardiology, adds up to the studies that shed light upon this issue and calls for an appeal to the journals and higher medical organizations to take up responsibilities by creating policies and programs that globally encourage and support women academically and financially to represent more in the scientific fields, researches, and publications.

Limitations

Firstly, the articles included in the study were sourced from PubMed only. By searching other indexing sites, more articles could have been analyzed. Furthermore, the present study only analyzed the first authors; analysis of all authors might have shed more insight regarding gender trends.

While other studies used Namsor with a considerable amount of success, we must acknowledge that the application programming interface is not 100% accurate.

## Conclusions

In conclusion, this research study reveals that although there is a rising trend of females in lead authorship roles within the field of academic publications related to WPW syndrome, it remains evident that there is a significant gender gap, with male researchers still outnumbering their female counterparts. The authors opine that efforts are needed to address this disparity and create a more balanced representation of genders in lead authorship positions. Promoting equal opportunities through targeted initiatives such as academic credits and providing support for female researchers will be instrumental in closing the gender gap and fostering a more inclusive research environment.

## References

[1] Jagsi R, Guancial EA, Worobey CC (2006). The "gender gap" in authorship of academic medical literature--a 35-year perspective. N Engl J Med.

[2] Chhabra L, Goyal A, Benham MD (2023). Wolff-Parkinson-White syndrome.

[3] Berry VA (1993). Wolff-Parkinson-White syndrome and the use of radiofrequency catheter ablation. Heart Lung.

[4] Morscher JH (1992). Wolff-Parkinson-White syndrome. AACN Clin Issues Crit Care Nurs.

[5] Asghar M, Usman MS, Aibani R (2018). Sex differences in authorship of academic cardiology literature over the last 2 decades. J Am Coll Cardiol.

[6] NamSor NamSor (2023). Namsoramsor, name checker for gender, origin and ethnicity determination. https://namsor.app/.

[7] Foley C, Harewood G, Benz E, Higgins L, Gibbons E, Kelly S, Cheriyan D (2022). Gender equality in academic gastroenterology: a review of gastroenterology literature over four decades. Ir J Med Sci.

[8] Amankwah N, Park M, Gu A, Choi BG (2018). Trends in authorship demographics for manuscripts published in the American Journal of Cardiology. Am J Cardiol.

[9] Morgan R, Lundine J, Irwin B, Grépin KA (2019). Gendered geography: an analysis of authors in The Lancet Global Health. Lancet Glob Health.

[10] Ravi K, Bentounsi Z, Tariq A (2021). Systematic analysis of authorship demographics in global surgery. BMJ Glob Health.

[11] Baobeid A, Faghani-Hamadani T, Sauer S (2022). Gender equity in health research publishing in Africa. BMJ Glob Health.

[12] Jian Y, Zhu D, Zhou D (2022). ARIMA model for predicting chronic kidney disease and estimating its economic burden in China. BMC Public Health.

[13] Rickard M, Hannick JH, Blais AS, Wang J, Santos JD, Lorenzo AJ (2020). Female authorship publishing trends and forecasting in pediatric urology: are we closer to gender equality?. Urology.

[14] Ross MB, Glennon BM, Murciano-Goroff R, Berkes EG, Weinberg BA, Lane JI (2022). Women are credited less in science than men. Nature.

[15] Richter KP, Clark L, Wick JA (2020). Women physicians and promotion in academic medicine. N Engl J Med.

[16] Rimmer A (2021). Two thirds of female cardiologists have experienced discrimination, survey finds. BMJ.

[17] Nakayama A, Morita H, Komuro I (2020). Female cardiologists in Japan. Int J Qual Health Care.

